# Transposon activity and nucleotide triplet instability: new perspectives on their potential interplay in brain disorders

**DOI:** 10.3389/fnins.2025.1617315

**Published:** 2025-09-16

**Authors:** Giuseppe Pepe, Marianna Storto, Alba Di Pardo, Vittorio Maglione

**Affiliations:** IRCCS Neuromed, Pozzilli (IS), Italy

**Keywords:** LINEs, SINEs, transposon activity, repeat instability, brain diseases

## Abstract

Genomic instability is a key feature of many neurological disorders, with transposon activation and nucleotide triplet repeat instability playing critical roles. Transposons, which are also referred to as mobile genetic elements, have the potential to destabilize the genome and interfere with gene expression. Conversely, changes in nucleotide triplet sequences, such as expansions or contractions, can lead to the production of abnormal proteins or nonfunctional RNAs. In this perspective, we discussed the intricate relationship between these two forms of genomic instability and their influence on brain disorders. We analyzed the molecular mechanisms that contribute to these phenomena, the shared regulatory systems that govern them, and their role in neurological conditions. Additionally, we provided some insights into the development of potential therapies for brain disorders linked to these genomic alterations.

## Graphical abstract

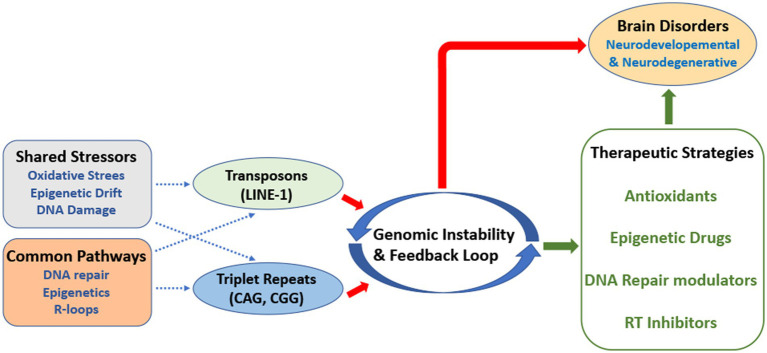

## Introduction

Neurological disorders include a wide range of conditions, often associated with complex genetic and epigenetic changes. The intricate relationship between transposon activity and nucleotide triplet repeat instability represents an intriguing area of research, with the potential to reveal mechanisms that may contribute to disease progression.

Transposons were once considered “junk DNA,” however, they are now recognized as active elements that shape genomic structure and gene expression, playing crucial roles in health and disease (Cordaux and Batzer, 2009). LINE-1 (long interspersed nuclear element-1) elements play an important role during brain development (Suarez et al., 2018). However, while they contribute to neuronal diversity, they also carry risks of genomic instability, highlighting their multifaceted role in brain physiology (Suarez et al., 2018).

In the context of brain disorders, retrotransposons have been implicated in various pathological processes (Zhang et al., 2025). In particular, LINE-1 elements may be reactivated in aging neurons and under stress conditions, leading to DNA damage and transcriptional dysregulation (Gorbunova et al., 2021; Della Valle et al., 2025). Oxidative stress may trigger LINE-1 activation in neuronal models, causing DNA damage and genomic instability (Ravel-Godreuil et al., 2021).

Nucleotide triplet repeat instability, characterized by the abnormal expansion or contraction of specific DNA sequences, is another genomic phenomenon associated with a number of neurodegenerative and psychiatric disorders (La Spada, 1997). Trinucleotide repeat expansions lead to transcriptional dysregulation, toxic RNA foci, and protein aggregation, which represent molecular hallmarks in the disease pathology (Everett and Wood, 2004; Sicot and Gomes-Pereira, 2013; Schwartz et al., 2021).

The interplay between transposon activity and nucleotide triplet repeat instability may be modulated by shared cellular stressors and regulatory pathways.

Oxidative stress, for instance, is a common trigger that can either activate LINE-1 retrotransposition or exacerbate triplet repeat expansions, through replication stress (Van Meter et al., 2014; Chatterjee et al., 2015; Martins et al., 2021; Ravel-Godreuil et al., 2021). Similarly, deficiencies in DNA repair system have been implicated in both repeat expansion and the mobilization of retrotransposons (Mirkin, 2007; Levin and Moran, 2011; Zhao and Usdin, 2015; Mendez-Dorantes and Burns, 2023). This highlights the interconnection between these types of genomic instabilities.

This perspective aims at unraveling the complex mechanisms underlying these processes, with a focus on their possible crosstalk and the potential pathways that could amplify their effects in brain disorders. Specifically, we argue that these two forms of instability are not merely parallel phenomena; they may engage to each other in a synergistic and deleterious interplay that may ultimately accelerate neuronal dysfunction. By exploring their contributions to brain diseases, we also aimed at highlighting some potential therapeutic strategies.

Understanding this interplay will be crucial for advancing our knowledge of neuronal dysfunction and for improving the development of therapeutic strategies.

## Transposon instability in brain health and disease

LINE-1 elements, constituting approximately 17% of the human genome, are retrotransposons capable of replicating and inserting themselves into new genomic locations (Richardson et al., 2014). Their activity is essential during neurodevelopment, where they are a major driver of neuronal genomic mosaicism (Richardson et al., 2014; Upton et al., 2015).

This mosaicism, characterized by diverse genomic configurations among neurons, is hypothesized to underlie individual neuronal functions and adaptability (Richardson et al., 2014; Faulkner and Garcia-Perez, 2017). Such a genomic diversity is crucial for cognitive processes, including learning and memory formation (Bachiller et al., 2017).

While, this activity promotes genomic innovation and adaptation, it may introduce risks, such as insertional mutagenesis and chromosomal instability, that can potentially compromise neuronal integrity (Richardson et al., 2014; Mendez-Dorantes et al., 2024).

The regulation of transposon activity is predominantly regulated by epigenetic mechanisms, including DNA methylation and histone modifications (Freeman et al., 2022; Lanciano et al., 2024). During early development, these mechanisms may act as crucial regulators of transposon activity to maintain genomic stability. Thus, any defect in such mechanisms, with consequent aberrant retrotransposon activity in neuronal precursor cells (NPCs; Jönsson et al., 2019), may be eventually linked to neurodevelopmental disorders such as Schizophrenia and Autism Spectrum Disorders (ASDs; Suarez et al., 2018; Jönsson et al., 2020). For instance, in schizophrenia, LINE-1 mobilization is associated with increased transposition in the neuronal genome (Bundo et al., 2014). Similarly, although the precise underlying mechanisms remain to be fully elucidated, ASDs are characterized by elevated levels of LINE-1 (Shpyleva et al., 2018) which has been suggested to contribute to defective neurodevelopmental processes (Evans and Erwin, 2021).

On the other hand, pathological age-related epigenetic decline (Li and Tollefsbol, 2016) may lead to hypomethylation of LINE-1 sequences, thereby increasing transposition events (Erwin et al., 2014; Senapati et al., 2023; Yang et al., 2023). This decline is particularly evident in neurodegenerative diseases (Brulé et al., 2025), where global hypomethylation, occurring in the adult brain, may potentially coincide with heightened transposon activity.

In this context, dysregulated transposon activity has been identified as a significant contributor to neurodegenerative disorders (Ochoa Thomas et al., 2020). Alzheimer’s disease displays increased LINE-1 activity that could compromise neuronal genome integrity, exacerbating disease progression (Guo et al., 2018; Ochoa Thomas et al., 2020; Roy et al., 2024). In Amyotrophic Lateral Sclerosis (ALS) models, LINE-1 activation has been linked to neuroinflammatory processes (Takahashi et al., 2022), while, in Parkinson’s disease neurons, translocation of LINE ORF1p induces nuclear envelop alterations (Martin, 2006; Znaidi et al., 2025).

## Nucleotide triplet repeat instability in brain disorders

Nucleotide triplet repeat instability plays a pivotal role in several brain disorders (Depienne and Mandel, 2021). Among these conditions, Huntington’s disease and Fragile X syndrome exemplify neurodegenerative and neurodevelopmental disorders, respectively (Salcedo-Arellano et al., 2020; Depienne and Mandel, 2021).

Nucleotide triplet repeat instability arises from different mechanisms like DNA replication slippage, defective mismatch repair, and oxidative DNA damage (Mirkin, 2007; Castel et al., 2010; Li and Bonini, 2010; McMurray, 2010).

Either expansions or contractions of specific repeat sequencing (Castel et al., 2010) can occur in both dividing cells and post-mitotic neurons due to the limited DNA repair capacity that may intensify instability over time (Konopka et al., 2022). In Huntington’s disease this leads to a phenomenon known as CAG repeat somatic instability (Cattaneo et al., 2025).

Expanded trinucleotide repeats can disrupt gene transcription, RNA splicing, and translation, leading to significant neuronal dysfunction (Castel et al., 2010; Li and Bonini, 2010; Schwartz et al., 2021). Moreover, repeat expansions can sequester vital RNA-binding proteins, like TDP-43, inducing neurodegeneration (Sun et al., 2023).

Furthermore triplet repeat expansions can affect chromatin dynamics (Frisch et al., 2001; Dion and Wilson, 2009; Nageshwaran and Festenstein, 2015) and change histone acetylation and methylation patterns (Kumari and Usdin, 2009; Wei et al., 2017), potentially modifying the transcriptional landscape of the neurons. In this context, CGG triplet expansions, associated with Fragile X Syndrome, have been linked to histone hypoacetylation and chromatin condensation, which contributes to the inactivation of FMR1 gene (Coffee et al., 2002).

## The potential mechanistic interplay between transposons and triplet repeats: a fascinating hypothesis

The pathological impact of these instabilities is age- and cell-type dependent. Transposon activation contributes to neuronal diversity during early neurodevelopment and is largely suppressed in mature neurons (Zhang et al., 2025). Its pathological reactivation occurs under age-related epigenetic erosion, oxidative stress, or neurodegenerative processes in post-mitotic neurons (Ochoa Thomas et al., 2020; Gorbunova et al., 2021).

Conversely, triplet repeat instability can originate from errors during DNA replication in developing cells (germline or somatic) that continues to accumulate in non-dividing neurons throughout life making neurons particularly vulnerable (McMurray, 2010; Konopka et al., 2022; Cattaneo et al., 2025). This may create a scenario where both instabilities can converge in aging and in the diseased neurons, potentially exacerbating genomic stress.

This mechanistic interaction between transposon activity and nucleotide triplet repeat instability, is a fascinating potential event which may reveal that these two forms of genomic instability may influence each other, thereby amplifying their deleterious effects.

### Oxidative stress as potential shared mechanism between transposon activation and triplet instability

Oxidative stress may influence both transposon activity and triplet repeat instability (Giorgi et al., 2011; La Rosa et al., 2020). It may induce LINE-1 activation in neuronal models (Giorgi et al., 2011) and reduce global DNA methylation and disrupt histone modifications (Niu et al., 2015; García-Guede et al., 2020), conceivable creating a permissive chromatin environment for LINE-1 activation.

Additionally, oxidative lesions of CAG repeats promote repeat expansions in Huntington’s disease models, suggesting a direct link between triplet instability and oxidative stress (Kovtun et al., 2007).

These findings suggest that oxidative stress could independently and synergistically activate mechanisms that may exacerbate genomic instability.

A bidirectional relationship between oxidative stress and genomic instability may generate a feedback loop involving transposon activity and triplet repeat expansions. For instance, ROS-induced DNA breaks may activate LINE-1 transposition, which in turn could likely introduce additional DNA damage. On the other hand, the generation of oxidative stress, in nucleotide triplet diseases, may create a self-sustaining loop of oxidative damage and DNA instability, which could also affect retrotransposon activity.

### Shared pathways in DNA repair system

Transposons and triplet repeats might compete for or interfere with common pathways involved in DNA repair.

Repeat expansions are often linked to DNA repair pathways (McMurray, 2010; Schmidt and Pearson, 2016; Lai et al., 2020). During their active transposition phases, transposons may affect the same repair pathways to support their insertion and stabilization within the genome (Gasior et al., 2006). DNA repair pathway, crucial for correcting replication errors, may be intimately involved in both processes. Proteins like MLH1, MSH2, MSH3 (component of DNA Mismatch Repair-MMR-system) and FAN1 (DNA repair nuclease) are key drivers of somatic CAG repeat expansion (Schmidt and Pearson, 2016; Deshmukh et al., 2021; Bunting et al., 2025). Concurrently, when transposable elements are highly active, their retrotransposition creates DNA double-strand breaks that could potentially hijack the DNA repair machinery (Morrish et al., 2002; Gasior et al., 2006; Mendez-Dorantes and Burns, 2023; Dias et al., 2025).

The condition in which DNA repairing is highly required, may theoretically make triplet repeats more susceptible to instability and expansion.

### RNA-mediated mechanisms

An interesting hypothesis is represented by the possibility that RNA may mediate a possible interaction between transposons and triplet repeats.

Triplet repeats can be associated with the formation of RNA–DNA hybrid structures (R-loops), which could potentially interfere with the regulation of gene expression and with DNA repair system and genome stability (Wulfridge and Sarma, 2024). Such a structures have been identified in brain disorders including neurodegenerative diseases (Li et al., 2025).

Interestingly, the existence of LINE1 transposition-derived R-loops has been recently shown (Paul et al., 2025). On the other hand, it has been also proposed that elevated R-loops may reactivate retroelements in some pathological conditions (Lim et al., 2015).

This highlights a potential R-loop-mediated cross talk between trinucleotide repeats and retrotransposons, able to exacerbate the instability of both elements and interfere with genome stability.

### Epigenetic interactions between transposons and triplet repeats

Epigenetic modifications, such as DNA methylation and histone modifications are essential in regulating the activities of both transposons and triplet repeats (Dion and Wilson, 2009; Protasova et al., 2021), suggesting a possible indirect interaction, mediated by changes in the epigenetic landscape.

For instance, DNA methylation is a well-documented mechanism that silences transposons, but it may also impact the stability of triplet repeat sequences (Dion and Wilson, 2009; Essebier et al., 2016; Jönsson et al., 2019; Poeta et al., 2020; Lanciano et al., 2024).

On the other hand, activation of transposable elements could themselves modify chromatin organization, DNA methylation state and histone acetylation at their insertion points (Hancks and Kazazian, 2016).

In this scenario, it is plausible that such epigenetic changes can even extend to nearby regions of the genome, possibly altering the dynamics of replication and repair processes for adjacent triplet repeats. In the case of Fragile X syndrome, the expanded CGG repeats, found in the FMR1 gene, correlate with changes in chromatin structure, which include heightened DNA methylation and repressive histone modifications (Yudkin et al., 2015).

These findings underscore the important, although indirect, role that transposons could play in shaping the epigenetic landscape of triplet repeats, potentially affecting gene expression and genome stability.

## Therapeutic opportunities: evidence from Huntington’s disease models

Huntington’s disease (HD) is a rare, inherited neurodegenerative disease that affects both the brain and the body, and worsens over time. The expanded CAG nucleotide repeat sequence in HTT gene, leads to the production of an abnormal protein (huntingtin) which gains new toxic functions (Tabrizi et al., 2020).

Instability of this triplet repeat sequence, which expands over generations, is characteristic of HD (Aziz et al., 2008), and is associated with neuronal CAG repeat mosaicism (Cattaneo et al., 2025).

Activation of retroelements has been reported in HD pre-clinical models as well as in human patients (Casale et al., 2021; Floreani et al., 2022).

Understanding how transposon instability relates to triplet repeat expansions could lead to new therapeutic strategies, however, tackling both types of instability at the same time represents unique challenges. Therefore, current strategies often focused on these instabilities separately.

Targeting DNA repair proteins (e.g., MSH3) was effective in reducing somatic expansions in patient-derived iPSC neurons (Bunting et al., 2025). Coherently, FAN1 expression stabilized CAG repeats in HD cell models (Goold et al., 2019).

Furthermore, evidence in a fly model of HD demonstrated elevated transposable element (TE) expression and mobilization, leading to neurodegeneration (Casale et al., 2021). The inhibition of TE mobilization, by reverse transcriptase (RT) inhibitors, rescued disease phenotypes, indicating a potential pathogenic role for TEs, and suggesting a potential therapeutic target for the disease (Casale et al., 2021).

Hypothetically, some approaches could be applied for both transposons and expanded triplets. For example, strategies aimed at targeting oxidative stress, which influences both instabilities (Giorgi et al., 2011; La Rosa et al., 2020), could offer a promising approach to mitigating their possible combined activity. In this context, although not yet investigated, the effect of the antioxidant agent N-acetylcysteine in HD models (Wright et al., 2015, 2016), could potentially address the possible interplay of transposon and triplet repeat instabilities.

On the other hand, strategies based on epigenetic regulation, which has shown a therapeutic potential in HD models (Hyeon et al., 2021; Dai et al., 2024), have been reported to modulate murine retrotransposons and chromatin structure (Brunmeir et al., 2010; Lennartsson et al., 2015).

## Discussion

In this perspective, we hypothesized that regulatory pathways, involving chromatin remodeling, DNA repair system and epigenetic marks could regulate both transposon activity and triplet repeat instability. We posit that these phenomena are likely interconnected to each other and create a vicious cycle of genomic damage in vulnerable neurons. Altered histone modifications in neurons, for example, may help activate transposons, while also destabilizing triplet repeats, promoting a greater genomic instability.

On the other hand, environmental triggers, such as oxidative damage may also contribute to generate an ideal “substrate” to maintain this combined instability.

Shared regulatory elements may act as master regulators, timing the activation of transposons and triplet repeats and possibly synchronizing these mechanisms.

Understanding how these factors can favor the possible interaction between transposon activity and triplet repeat instability could pave the way for a better understanding of disease molecular mechanisms and for the development of genome-targeted therapeutic strategies.

In this context, the temporal dynamic may be a critical consideration. While retrotransposon activity can be beneficial during neurodevelopment, its pathological reactivation in post-mitotic neurons, a cell type with limited DNA repair capacity, may be especially destructive. Theoretically, any early activation of transposable elements, occurring in the developing brain, may influence triplet instability and related disease phenotypes in adult neurons.

As shown in the Table 1, all the disease conditions reported, spanning both neurodevelopmental and neurodegenerative disorders, demonstrate the potential relevance of these concepts across the lifespan (developing and adult brain) and across different cell types (NPCs and neurons).

**Table 1:** Neurological disorders associated with genomic instability.

Neurological disorder	Genomic instability	Specific element/repeat	Impacted cell type (Stage)	Implicated mechanism/pathological role
Schizophrenia	Transposon-mediated	LINE-1 (Jönsson et al., 2019; Bundo et al., 2014)	NPCs (Neurodevelopment); Neurons (Adult Brain)	Increased transposition in the neuronal genome, linked to aberrant retrotransposon activity in NPCs
Autism Spectrum Disorders	Transposon-mediated	LINE-1 (Shpyleva et al., 2018; Jönsson et al., 2020)	NPCs (Neurodevelopment)	Elevated levels of LINE-1 contributing to defective neurodevelopmental processes
Alzheimer’s Disease	Transposon-mediated	LINE-1 (Guo et al., 2018; Roy et al., 2024)	Neurons (Aging/Disease)	Increased LINE-1 activity exacerbating disease progression and compromising neuronal genome integrity
Amyotrophic Lateral Sclerosis	Transposon-mediated	LINE-1 (Takahashi et al., 2022)	Neurons (Adult brain)	Linked to neuroinflammatory processes
Parkinson’s Disease	Transposon-mediated	LINE ORF1p (Znaidi et al., 2025)	Neurons (Aging/Disease)	Translocation of LINE ORF1p induces nuclear envelop alterations
Fragile X Syndrome	Triplet Repeat-mediated	CGG (Coffee et al., 2002)	Neurons (Neurodevelopment)	Histone hypoacetylation and chromatin condensation, contributing to FMR1 gene inactivation
Huntington’s Disease	Triplet Repeat-mediated	LINE-1/CAG (Floreani et al., 2022; Casale et al., 2021)	Neurons (Adult Brain)	Production of an abnormal protein (huntingtin) and somatic instability and mosaicism. Activation of retroelements has been reported in HD pre-clinical models as well as in human patients. Inhibition of TE mobilization, rescued disease phenotypes in a fly model of disease

Moreover, the possibility that both form of genome instability might share some mechanistic elements suggests a new therapeutic paradigm.

To our perspective, future therapeutic efforts could focus on the master regulatory pathways that govern both genome instabilities, rather than targeting them separately. In this contest, drugs aimed at restoring global epigenetic repression or boosting the efficiency of shared DNA repair pathways could potentially offer a synergistic effective therapeutic approach.

## Data Availability

The original contributions presented in the study are included in the article/supplementary material, further inquiries can be directed to the corresponding author.
